# Cuticle Modifications and Over-Expression of the Chitin-Synthase Gene in Diflubenzuron-Resistant Phenotype

**DOI:** 10.3390/insects13121109

**Published:** 2022-11-30

**Authors:** Valentina Lucchesi, Lorenzo Grimaldi, Valentina Mastrantonio, Daniele Porretta, Letizia Di Bella, Tania Ruspandini, Martino Luigi Di Salvo, John Vontas, Romeo Bellini, Agata Negri, Sara Epis, Silvia Caccia, Claudio Bandi, Sandra Urbanelli

**Affiliations:** 1 Department of Environmental Biology, Sapienza University of Rome, 00185 Rome, Italy; 2 Department of Earth Sciences, Sapienza University of Rome, 00185 Rome, Italy; 3 Department of Biochemical Sciences “A. Rossi Fanelli”, Sapienza University of Rome, 00185 Rome, Italy; 4 Department of Crop Science, Pesticide Science Lab, Agricultural University of Athens, 11855 Athens, Greece; 5 Institute of Molecular Biology and Biotechnology, Foundation for Research and Technology Hellas, P.O. Box 1385, GR-711 10 Heraklion, Greece; 6 Medical and Veterinary Entomology, Centro Agricoltura Ambiente “G. Nicoli”, 40014 Bologna, Italy; 7 Department of Biosciences, University of Milan, 20122 Milan, Italy; 8 Pediatric CRC “Romeo ed Enrica Invernizzi”, University of Milan, 20122 Milan, Italy

**Keywords:** chitin synthesis inhibitors, chitin synthase, insect vectors, insecticide resistance, mosquitoes, pest control, resistant phenotype

## Abstract

**Simple Summary:**

Insect pests are a major problem worldwide and their control represents an urgency for human well-being. Insecticides acting as chitin-synthesis inhibitors (CSIs) are one of the major tools used to control these insects, but resistance is evolving in many species against several chemical classes, such as oxazolines and benzoylureas. Genetic studies showed that resistance is associated with point mutations in position 1043 of the chitin-synthase 1 gene (*chs*1), which change an isoleucine into phenylalanine, leucine or methionine (i.e., I1043F; I1043L; I1043M, respectively). Here, we studied the molecular and phenotypic characteristics of the homozygous I1043M strain in the mosquito *Culex pipiens* by comparing susceptible and resistant individuals. Our results showed that resistant mosquitoes have a striking level of resistance when exposed to the CSI diflubenzuron. Furthermore, cuticle modifications and the over-expression of the *chs*1 gene were detected in resistant *Cx. pipiens*, which are associated and, thus, likely contribute to resistance, as well as to the intensity of the resistant phenotype. Since mutations on the *chs*1 gene are conserved among different arthropod pest species, our results could be valuable to understand CSI resistance not only in mosquitoes, but also in a wider perspective.

**Abstract:**

Insecticide resistance is a major threat challenging the control of harmful insect species. The study of resistant phenotypes is, therefore, pivotal to understand molecular mechanisms underpinning insecticide resistance and plan effective control and resistance management strategies. Here, we further analysed the diflubenzuron (DFB)-resistant phenotype due to the point-mutation I1043M in the chitin-synthase 1 gene (*chs*1) in the mosquito *Culex pipiens*. By comparing susceptible and resistant strains of *Cx. pipiens* through DFB bioassays, molecular analyses and scanning electron microscopy, we showed that the I1043M-resistant mosquitoes have: (i) a striking level of DFB resistance (i.e., resistance ratio: 9006); (ii) a constitutive 11-fold over-expression of the *chs*1 gene; (iii) enhanced cuticle thickness and cuticular chitin content. *Culex pipiens* is one of the most important vector species in Europe and the rapid spread of DFB resistance can threaten its control. Our results, by adding new data about the DFB-resistant phenotype, provide important information for the control and management of insecticide resistance.

## 1. Introduction

Harmful insect species represent a long-standing problem affecting human activities. In agriculture, pests have often been documented to reduce crop production, by destroying plant biomass and accelerating the decay process [1]. Likewise, in terms of human health, insect vectors have a profound effect, by spreading and transmitting pathogens that cause human and animal diseases [2].

Until the end of the last century, chemical insecticides have been the most efficient method to control pest species and mitigate their effects worldwide. However, the development of insecticide resistance has strongly hampered their efficacy and the adoption of biorational approaches is increasing. The intensive use of insecticides, indeed, has selected alleles conferring resistance in wild populations, overwhelming the effect of a wide range of compounds in several target species [3]. As reported in previous studies, since the 1950s, the number of insecticides for which one or more species evolved resistance has progressively increased and, recently, it exceeded 300 compounds [4].

Chitin synthesis inhibitors (CSIs) have been among the most important compounds employed in recent pest-management programs. These molecules are released by spraying or as larvicides in species breeding sites and act against insects and mites by inducing malformations of the cuticle and abortive molting [5,6]. Recently, the development of insecticide resistance against CSIs has been documented in several harmful pest species, such as *Tetranychous urticae*, *Plutella xylostella* and *Cydia pomonella* [7,8,9]. Further genetic studies showed that resistance to CSIs was associated with non-synonymous mutations in the chitin-synthase 1 gene (*chs*1) [10,11], a pivotal β-glycosyltransferase acting during cuticle formation [5,6]. In 2015, the same mutations in the *chs*1 gene were described for the first time in mosquitoes, in the species *Culex pipiens* [12].

*Cx. pipiens* is a widespread species in European regions, where it is the major vector of West Nile virus (WNV) [13,14]. Recently, the occurrence of populations resistant to the CSI diflubenzuron (DFB) was detected in Italian populations [12]. Additional genetic studies showed that resistant individuals harboured point mutations at position I1043 of the *chs*1 gene, an equivalent position to the oxazolines and benzoylureas (BPUs, including DFB) resistance mutations detected in *T. urticae* and *P. xylostella* [12]. In susceptible *Cx. pipiens*, nucleotide sequences code for isoleucine amino acid at the I1043 position, while resistant mosquitoes harbour mutated sequences coding for leucine, methionine or phenylalanine (hereafter I1043L, I1043M and I1043F, respectively) [12,15]. Since its first detection in the Emilia-Romagna region, some aspects about DFB resistance have been elucidated. Genome-editing studies have functionally validated the three resistant mutations in laboratory strains of *Drosophila melanogaster* using CRISPR/Cas9 technology and showed that they confer different levels of resistance (i.e., resistance ratio (RR) > 15,000 for I1043M and I1043F; RR >20 for I1043L) [11,12]. Furthermore, monitoring surveys showed that resistant mutations occur also beyond the Italian boundaries, in Turkish and French populations [15,16]. More recently, phylogenetic analyses also showed that the I1043F, I1043M and I1043L mutations originated multiple times and independently in the areas where they occur [17]. To date, although important genetic and functional information has been collected about DFB resistance, few studies have directly explored the phenotypic effects of DFB resistance in *Cx. pipiens* strains [12].

In this paper, we aimed to contribute to this issue by investigating the molecular and phenotypic characteristics of a resistant strain of *Cx. pipiens,* homozygous for the I1043M mutation. The I1043M mutation is particularly interesting among the three resistant alleles because it has a wide geographic distribution and confers high levels of resistance in other species [11]. Therefore, starting from field samples, we established susceptible and resistant laboratory colonies of *Cx. pipiens* and compared them to assess: (i) the resistance level of the mutated I1043M strain; (ii) the relative gene expression of the chitin-synthase 1 gene, the target of diflubenzuron; (iii) the occurrence of modifications in cuticle thickness and chitin content.

## 2. Materials and Methods

### 2.1. Establishment and Maintenance of Resistant and Susceptible Mosquito Colonies

Susceptible and resistant colonies of *Cx. pipiens* (hereafter, *Cp*-S and *Cp*-R-I1043M) were established starting from wild samples collected in the Emilia-Romagna region during summer 2020. Based on previous results about the frequency of resistant and wild-type alleles in *Cx. pipiens* populations [15,18], immature mosquitoes were collected in the urban areas of Forlì and Parma to establish the resistant and susceptible colonies, respectively. In each town, at least five breeding sites were sampled to have a representative sample of the population. Once collected, mosquito larvae were transferred in the Evolutionary Ecology laboratory (Sapienza University, Rome, Italy) and morphologically and genetically identified as *Cx. pipiens* [19] For genetic identification, a fragment of the mitochondrial gene Cytochrome Oxidase I (COI) was amplified and sequenced by using previously designed primers (CxCOI_F 5′-AAAAAGATGTATTTAAATTTCGGTCTG-3′; CxCOI_R-5′-TGTAATTGTTACTGCTCATGCTTTT-3′).

To realize the resistant homozygous I1043M colony, collected mosquitoes were exposed to DFB, using an LD_50_ dose previously estimated in field populations of *Cx. pipiens* from Ravenna (i.e., 0.06 mg/L, [12]). After DFB exposure, survivors were reared until adulthood and left to mate. Since in previous years the I1043L- and I1043F-resistant alleles were also found in resistant populations, including Forlì [15,18], a genetic identification of individuals was also performed to maintain only the I1043M allele. Specifically, after female oviposition, single egg rafts were allowed to hatch and the offspring was genetically analysed using a PCR-based assay on the *chs*1 gene, specific for the I1043M mutation [12]. Only homozygous depositions for the I1043M allele were retained for the colony establishment, while the others were discarded. For the susceptible colony, since resistant alleles have never been found in Parma [15,18], all collected mosquitoes identified as *Cx. pipiens* by morphology were used to establish the colony. The susceptible genotype of the individuals was, however, verified by sequencing a fragment of the *chs*1 gene spanning the codon where resistant mutations occur [12].

Both *Cp*-S and *Cp*-R-I1043M colonies were reared in a thermostatic chamber with standard conditions of temperature (26 ± 1 °C), relative humidity (70%) and photoperiod (16:8 h light: dark). Larval stages were daily fed with fish food (0.85 mg/larva) (Tetra^®^ Goldfish Granules). Adults were maintained in 45 × 45 × 45 cm Bugdorm© insect-rearing cages (Watkins & Doncaster, Leominster, UK) and daily fed with 10% sucrose solution and water ad libitum. To maintain the colonies, blood meal was provided to females by using the Hemotek membrane-feeding system (Hemotek Ltd.©, Blackburn, UK). Mosquitoes from 6th generation of both colonies were used for the current study. A PCR-based approach designed in *Cx. pipiens* and specific for the I1043M allele was used to check that the I1043M allele frequency at this generation was 100% [12].

### 2.2. DFB Bioassays

Bioassays with diflubenzuron (DFB) were performed according to the WHO international guidelines (WHO, 2016). DFB (PESTANAL, C_14_H_9_ClF_2_N_2_O_2_, Sigma-Aldrich S.r.l., Milan, Italy) was diluted in acetone (ACS reagent, 99.5%, Sigma-Aldrich, Milan, Italy) to prepare the 6 mg/mL stock solution. Then, the stock solution was diluted with water to make the test solutions. A range of five concentrations was selected and used to determine LD_50_ (the dose at which 50% of insects are dead). In particular, for the *Cp*-S strain the DFB concentrations tested were: 0.0005 mg/L, 0.002 mg/L, 0.01 mg/L, 0.1 mg/L and 0.3 mg/L, while for the *Cp*-R-I1043M, the concentrations 7.5 mg/L, 25 mg/L, 50 mg/L, 75 mg/L and 100 mg/L were used. Groups of twenty-five late 3rd-instar larvae of *Cx. pipiens* were placed in plastic cups with 100 mL of water and treated with DFB. Control tests treated with water and acetone were also included. All the experiments were performed in quadruplicate and three biological replicates were performed. Since CSIs, such as DFB, have a delayed action on treated individuals [6], the mortality rate was assessed every two or three days until all individuals were dead or emerged as adults. At the end of the experiments, the software Polo Plus [20] was used to estimate LDs and their 95% confidence intervals (CIs); then the LD_50_ values of susceptible and resistant strains were used to calculate the resistance ratio (RR = LD_50_ resistant strain/LD_50_ susceptible strain).

### 2.3. Expression Analysis of chs1 Gene

Gene expression analysis was performed to investigate and compare the constitutive expression of *chs*1 gene in resistant and susceptible *Cx. pipiens* mosquitoes. Total RNA was extracted from three pools of ten resistant and susceptible 4th-instar larvae using the TripleXtractor directRNA kit (GRiSP Research Solutions, Porto, Portugal), according to the manufacturer’s instructions. Once extracted, the amount and the quality of RNA were assessed using Qubit™ 3 Fluorometer (ThermoFisher Scientific, Waltham, MA, USA) and NanoDrop ND-1000 (Thermo Scientific, Waltham, MA, USA). cDNA was then synthesized from 200 ng of total RNA using the FIREScript Reverse Transcriptase (RT) kit (Solis Biodyne, Tartu, Estonia).

The cDNA was used as template for quantitative reverse transcription (RT)-qPCR. The RT-qPCR was performed using a QuantStudio^TM^ 5 Real-Time PCR System (Applied Biosystem, ThermoFisher Scientific) with the ExcelTaq™ 2X Fast Q-PCR Master Mix (SMOBIO Technology, Hsinchu, Taiwan), under the following conditions: 50 ng of cDNA; 300 nM of forward and reverse primers; 98 °C for 30 s, followed by 40 cycles of 98 °C for 15 s and 59 °C for 30 s; fluorescence was acquired at the end of each cycle and melting curve analysis was performed after the last cycle. Primers are reported in Table 1. Relative expression of the *chs*1 gene was calculated using the cycle threshold (Ct) value and the two endogenous genes (i.e., G6PDH and *18S ribosome RNA*). The expression levels of the susceptible mosquitoes were considered as the basal level (set equal to 1). The relative expression of resistant and susceptible individuals was reported as means ± standard deviation (SD) and compared using Student’s *t*-test (*p*-value < 0.05), as implemented in the software GraphPad Prism v.8.0.1 (GraphPad Software Inc., San Diego, CA, USA).

### 2.4. Cuticle Analysis

#### 2.4.1. Analysis of Cuticle Thickness by Scanning Electron Microscopy (SEM)

Cuticle observations of *Cx. pipiens* mosquitoes were performed through Scanning Electron Microscope (FEI Quanta 400, Diepoldsau, Switzerland) at the SEM Laboratory of the Department of Earth Science, Sapienza University of Rome (Rome, Italy). A total of 20 resistant and susceptible individuals from different developmental stages (5 4th-instar larvae; 5 pupae; 5 adult females; 5 adult males) was collected and frozen at −20 °C for subsequent analyses. Before SEM observation, total body length for larvae, cephalothorax width for the pupae and wing length for adults were measured and used as proxies for body size [23,24,25]. Then, individuals with similar size (i.e., differences in body length, cephalothorax width and wing length < 0.01 mm for larvae, pupae and adults, respectively) were chosen and used for subsequent SEM analysis.

According to Bhattacharya et al. [26], the samples were placed in a 1.5 mL Eppendorf tube (Eppendorf^®^, Milano, Italy) and first immersed in a fixative solution containing 2.5% glutaraldehyde and 4% paraformaldehyde in 0.1 M of phosphate buffer. After one hour, the samples were dehydrated in increasing concentrations of ethanol (30%, 50%, 70%, 80% and 90% 10 min each, and 95% for 40 min) and then maintained for 10 min in a mixture of 95% ethanol and isoamyl acetate (1:1) and for 15 min in pure isoamyl acetate. For chemical drying, the samples were immersed in hexamethyldisilane (HMDS) for 5 min. Finally, the samples were dried in a heater at 40 °C for 30 min. Transverse sections of the first abdominal segment were made with a scalpel under a stereomicroscope (Leica EZ4W, Milano, Italy) and attached to 12.5 mm SEM stubs using carbon tabs. The samples were then coated in gold using an Emitech K550X (Labtech, Heathfield, UK) sputter coater, with a routine cycle time for coating SEM samples with conductive coating (5–15 nm) of gold typically less than four minutes. The sections were examined in high-vacuum mode, using an accelerating voltage of 15 kV and pictures of the cuticle were taken at a resolution of 10 µm. Thicknesses of 30 points of the cuticle were measured in the SEM images for each individual using the software ImageJ, (National Institute of Mental Health, Rockville Pike, Bethesda, MD, USA) in order to obtain the mean cuticular thickness (Figure S1). The comparison between the cuticular thickness in resistant and susceptible individuals was performed with Mann–Whitney’s test using the software GraphPad Prism v.8.0.1.

#### 2.4.2. Quantification of Chitin Content

Pools of 4th-instar larvae of the *Cp*-S and *Cp*-R-I1043M strains were analysed to identify differences in chitin content. For both strains, larvae with similar size (i.e., differences in body length below 0.01 mm) were used to form the pools. To quantify the chitin content in both strains, an adaptation of the calcofluor staining method reported in Henriques et al. [27] was used. First, a 12.5 μg/μL colloidal chitin suspension was prepared starting from commercial chitin from shrimp shells (C7170, Sigma Aldrich, Milan, Italy) following a previously described methodology [28]. This suspension was used to build a standard curve relating the amount of chitin to the fluorescence intensity of the calcofluor white fluorescent brightener (Sigma Aldrich, Milan, Italy) bound to chitin (Figure S2). To this end, 0 to 6 μL of the colloidal chitin suspension was placed in 1.5 mL Eppendorf tubes to which 100 μL of a 1 mg/mL dimethyl sulfoxide (DMSO) dilution of the calcofluor was added. The samples were kept in the dark for 15 min to allow for binding between the dye and the chitin. After this, samples were centrifuged for 5 min at 12,000 rpm to remove the excess of dye, and then two washes were carried out with 300 μL of distilled water, taking care not to disturb the pellet on the bottom of the tubes. After washing, the pellets were resuspended in 200 μL of water and transferred to a black Costar 96 plate (Merk Life Science S.r.l., Milano, Italy) for fluorescence measurement on a TECAN INFINITE M200 fluorescence plate reader. (TECAN, Männedorf, Switzerland). Excitation was set at 355 nm, emission at 433 nm. Relative Fluorescence Units (RFUs) were recorded in recommended “optimal” mode of the MAGELLAN software (TECAN, Männedorf, Switzerland) attached to the instrument. The assay was linear in a range of 0 to 65 μg of chitin. Linear regression was applied to estimate the unknown amount of chitin in the tested samples by fitting the obtained RFU fluorescence to the standard curve.

Larval specimens of the *Cp-*S and *Cp*-R-I1043M strains were then analysed. Three pools of twenty 4th-instar larvae for both phenotypes were collected and kept at −20 °C prior to analyses. Once thawed, 200 μL of distilled water was added to each pool and the sample was extensively manually crushed with a 2 mL volume glass Potter (Vetro Scientifica s.r.l., Rome, Italy). The resulting homogenates were divided into eight tubes, each containing 25 μL of sample. Then 100 μL of the 1 mg/mL solution of calcofluor was added to every test tube and the samples were treated as previously described for the standard curve construction.

To assess the significance of the differences in chitin content between susceptible and resistant individuals, the t-test with Welch correction was used, as implemented in the software GraphPad Prism v.8.0.1.

## 3. Results

### 3.1. DFB Bioassay

The mortality data obtained performing bioassays with both susceptible and resistant mosquitoes well fitted the Probit dose–response model (test χ^2^, *p* > 0.05). In the *Cp-*S strain, the Probit analysis revealed an LD_50_ equal to 0.005 mg/L (95%, 0.002–0.008), while in the *Cp*-R-1043M strain, an LD_50_ value of 45.04 mg/L (95%, 13.06–60.66) was found (Table 2; Figure S3). Thus, the *Cp*-R-1043M strain showed considerable resistance to diflubenzuron, with a resistance ratio (RR_LD50_) = 9006-fold. A mortality never exceeding 10% was observed in the controls. 

### 3.2. Expression of chs1 Gene in Resistant and Susceptible Cx. pipiens Mosquitoes

**Figure 1:**
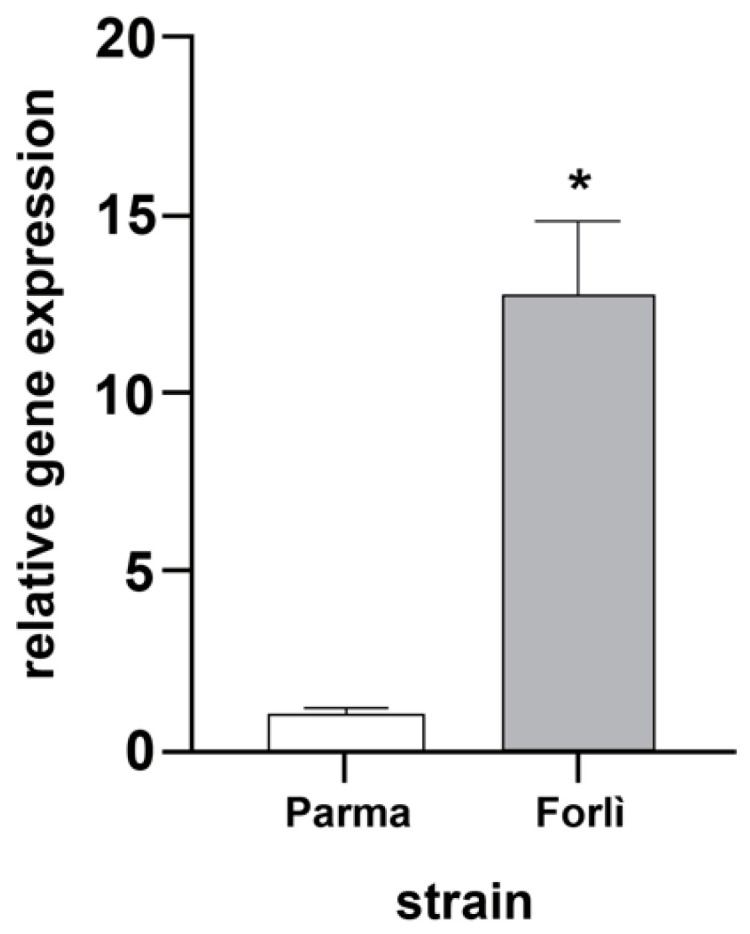
Analysis of *chs*1 relative expression in *Cx. pipiens*. Relative expression of the *chs*1 gene in the Parma (susceptible) and Forlì (resistant) strains. Susceptible individuals were used as basal level (equal to 1). The values reported are the mean ± standard errors (* *p* < 0.001, Student’s *t*-test).

The results of gene expression analysis are shown in Figure 1. Significant differences in constitutive expression of the *chs*1 gene were found between the two strains (*t*-test, *p*-value < 0.001). In particular, an 11-fold increase in *chs*1 transcripts was detected in the *Cp*-R-1043M samples compared to those of the *Cp*-S strain, suggesting a constitutive over-expression of this gene in resistant mosquitoes.

### 3.3. Cuticle Thickness

**Figure 2:**
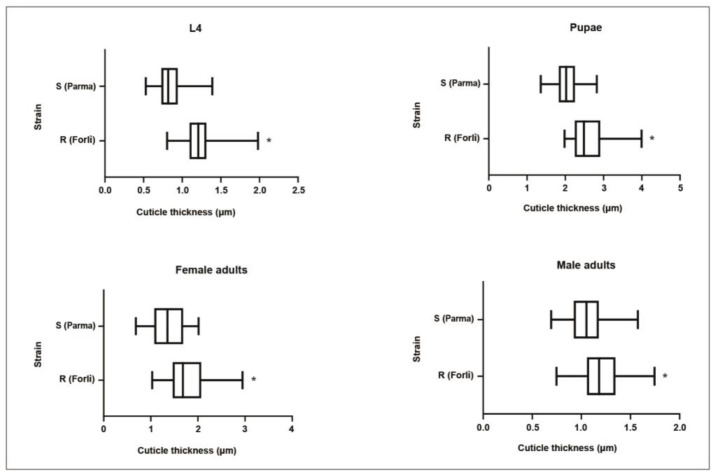
Analysis of cuticle thickness in *Cx. pipiens.* Cuticular thickness in susceptible and resistant mosquitoes at different life stages is shown. The boxes represent the 25% and 75% percentile and the black lines within the boxes indicate the medians; error bars correspond to the 10th and 90th percentiles. * *p* < 0.001 as determined by the Mann–Whitney test.

The SEM analysis of the cuticle thickness showed significant differences between *Cp*-R-1043M and *Cp*-S strains (Mann–Whitney Test, *p* < 0.001). Resistant mosquitoes had a thicker cuticle than susceptible mosquitoes at all life stages analysed (i.e., larvae, pupae and adults) (Table 3, Figure 2). Compared to the *Cp*-S strain, cuticle thickness in resistant mosquitoes showed an increase, ranging from 14% to 41%, observed in male adults and larvae, respectively. In both strains, among the life stages analysed, the higher value of cuticle thickness was observed in the pupal stage.

### 3.4. Quantification of Chitin Content

**Figure 3:**
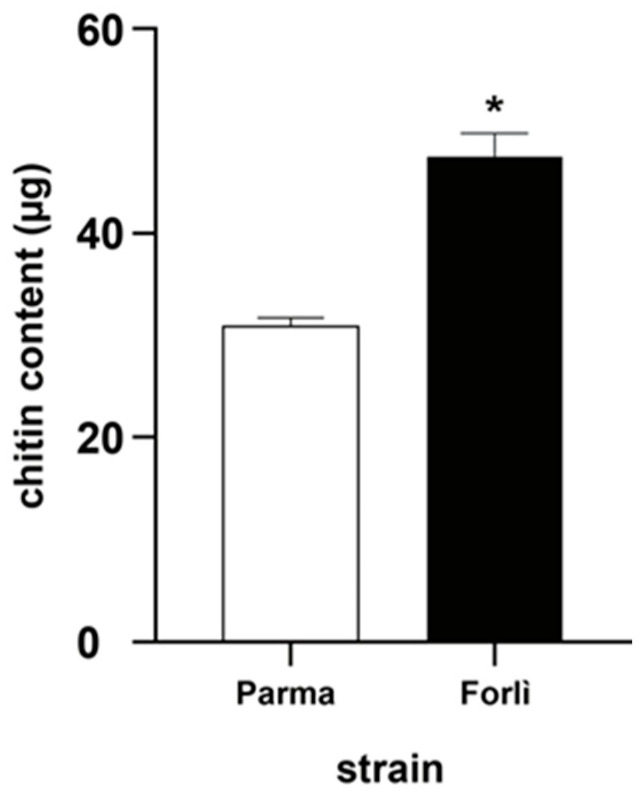
Analysis of chitin content in *Cx. pipiens*. Quantification of chitin content in susceptible and resistant mosquitoes. The values reported are the mean ± standard errors (* *p* < 0.001, Student’s *t*-test).

To estimate the amount of chitin content in susceptible and resistant larvae of *Cx. pipiens*, a fluorimetric approach using the calcofluor assay was used. As shown in Figure 3, a higher value of chitin content was observed in Cp-R-1043M individuals compared to the Cp-S strain. In particular, the mean value of the cuticle content observed in susceptible and resistant pools was 30.89 ± 1.02 µg and 47.77 ± 2.02 µg, respectively (i.e., 1.54 µg/larva and 2.39 µg/larva in Cp-S and Cp-R-1043M strain, respectively).

## 4. Discussion

DFB is one of the few larvicides still available to control harmful insect species, including *Cx. pipiens.* The occurrence of alleles conferring resistance to DFB has been recently documented in populations of *Cx. pipiens* sampled in the Emilia-Romagna region [12,15,18,29]. Field-monitoring studies showed that resistant alleles diffused across the region, becoming frequent in several provinces in Emilia-Romagna (i.e., relative frequency of I1043L, I1043M and I1043F up to 65%, 93% and 10%, respectively) [15,18]. Under these circumstances, studying resistant phenotypes is pivotal to re-organize effective control strategies. Here, by using different approaches, we studied the *Cp*-R-1043M strain of *Cx. pipiens* with the final aim to enhance our knowledge about the molecular and phenotypic characteristics associated with DFB resistance.

Larval bioassays were conducted on susceptible and resistant strains of *Cx. pipiens* to assess the level of DFB resistance. Our results showed that the LD_50_ estimated for the *Cp*-S and *Cp*-R-1043M strains was significantly different (Table 2), with mortality of our susceptible strain similar to that previously reported in other strains (i.e., LD_50_ of 0.002 mg/L) [12]. Importantly, our results revealed that the *Cp*-R-1043M strain was strikingly less susceptible than the *Cp*-S strain (RR = 9006). Previous genome-modification studies in *D. melanogaster* showed that the insertion of the I1043M mutation in a susceptible genetic background allowed mutated flies to survive to a very high dosage of DFB compared to the susceptible individuals (RR ranging from 2900 to 15,000) [11,12]. The high RR observed in our *Cx. pipiens*-resistant strain echoes the results obtained in model systems and further supports the strong impact that this resistance may have on the DFB performance in the field. Notably, the I1043M allele is rapidly expanding in the Emilia-Romagna region and mosquitoes with homozygous genotypes for the I1043M allele have been frequently observed in different provinces, also with high relative frequency [15,18]. Furthermore, this mutation has also been found in other regions across Europe, such as Turkey and France [15,16].

The analysis of the cuticle thickness and chitin content further showed unique characteristics of resistant mosquitoes compared to the susceptible strain. A thicker cuticle in resistant mosquitoes was observed by SEM, and this pattern was found consistent across all tested life stages, from larvae to adults. In addition, resistant larvae also showed an increased chitin content compared to susceptible larvae. Changes in cuticle thickness and composition have been reported in several arthropod species resistant to pyrethroids or organochlorine insecticides [30,31,32]. In mosquitoes, for example, an increase in cuticle thickness was observed in a pyrethroid-resistant strain of *Anopheles funestus* compared to susceptible individuals [30]. Similarly, in *Cx. pipiens pallens*, a thicker femur cuticle was found in resistant versus susceptible mosquitoes [33]. In all these documented cases, the observed cuticular changes were attributed to the over-expression of different genes encoding for cytochrome-P450s, cuticular proteins, laccases and ABC transporters, promoting an enriched deposition of some structural components within the cuticle, such as epicuticular lipids or cuticular proteins (i.e., leading to the so-called “penetration resistance”) [34].

The case of *Cx. pipiens* reported herein, to our best knowledge, is the first case documenting the occurrence of cuticle modifications in strains resistant to DFB. This observation, therefore, may act as a trailblazer to investigate if the mechanisms underpinning penetration resistance against pyrethroids, or organochlorines, can also contribute to DFB resistance, or if other molecular mechanisms are involved. Interestingly, gene expression analysis of the *chs*1 gene revealed an 11-fold over-expression of this gene in the *Cp*-R-1043M strain compared to the *Cp*-S mosquitoes. Chitin synthase is a pivotal enzyme in the formation of an insect cuticle because it catalyses the final steps of chitin synthesis, a major structural component of the cuticle [35]. In particular, this complex glycosyltransferase enables the elongation and translocation of nascent chitin chains across the plasma membrane, allowing their assemblage and deposition at the body surface [5]. The over-expression of the *chs*1 gene found in resistant *Cx. pipiens* mosquitoes could, therefore, explain the enhanced cuticle thickness and chitin content revealed by our analyses. Further studies integrating our results with data about gene expression of the main detoxifying families will be useful to support this hypothesis. Notably, if this hypothesis will be verified by new collected data, the resistance to DFB in *Cx. pipiens* would be an outstanding case study, where the same target gene (i.e., *chs*1) would drive both target-site resistance and cuticle modifications, reducing the intake of insecticide: the I1043M point mutation would affect the efficacy of DFB molecules by causing an aminoacidic change in the DFB target-site; the over-expression of the *chs*1 gene would positively affect the cuticle deposition.

Taken together, the observations in *Cx. pipiens* raise interesting implications for control and resistance management. The cuticular modifications observed in immature and adult stages could broaden the resistance of *Cx. pipiens.* Since enhanced cuticle thickness has been documented to reduce the penetration of several insecticide molecules [34], cases of cross-resistance could emerge in this species, with considerable consequences in resistance management and control practices. Furthermore, the cuticle changes observed in the *Cp*-R-1043M mosquitoes could affect the spreading of resistance in *Cx. pipiens*, by determining a fitness cost. The cuticle is a well-organized and conserved structure that is involved in several functions, such as water balance, perception of the environment, locomotion and chemical communication [36]. The modifications occurring in resistant mosquitoes could, therefore, affect all these biological functions, finally affecting the individual fitness. Since the occurrence of a fitness cost in resistant strains is one of the main variables affecting resistance diffusion, changes in cuticle structure and composition could significantly impact the speed of resistance spreading. Future studies, aiming to investigate if a fitness cost occurs in DFB-resistant phenotypes of *Cx. pipiens,* will be required to expand the evidence about the above issues, as well as to enhance our knowledge about the factors affecting insecticide resistance.

## Figures and Tables

**Table 1:** Primers pairs used in gene expression analyses. For each gene, the forward and the reverse primers are shown. HK = housekeeping gene.

Gene	Primer Sequences (5′-3′)	Fragment Length (bp)	Reference
G6PDH (HK)	F: CGCGCACGAGGAAAAGTACG R: GGTTTGCGGTCTTCCCAACC	131	[21]
18S (HK)	F: CGCGGTAATTCCAGCTCCACTA R: GCATCAAGCGCCACATATAGG	159	[22]
*chs*1	F: GCCTGTCTCCATCCGCAAG R: CCCAGGAGACGACGTTCAG	124	[12]

**Table 2:** Lethal doses observed in resistant and susceptible *Cx. pipiens* colonies. LD50 (95% CI) = Lethal dose of DFB required to kill 50% of population and its 95% confidence intervals; LD90 (95% CI) = Lethal dose of DFB required to kill 90% of population and its 95% confidence intervals; ^†^ Resistant Ratio.

Strain	LD50 (95% CI)	LD90 (95% CI)	RR_LD50_^†^
*Cp*_S	0.005 mgL^−1^ (0.002–0.008)	0.018 mgL^−1^ (0.012–0.031)	1
*Cp*_R-I1043M	45.03 mgL^−1^ (13.06–60.66)	85.81 mgL^−1^ (69.56–125.85)	9006

**Table 3:** Cuticle thickness in susceptible and resistant *Cx. pipiens* mosquitoes. The cuticle thickness is reported for all the life stages analysed and the values are expressed in μm. N = number of mosquitoes analysed for each strain. The values are reported as the mean ± standard errors (* *p* < 0.001, Mann–Whitney test).

Strain	N	Developmental Stages			
		L4 Larvae	Pupae	Male Adults	Female Adults
*Cp*_S	150	0.86 (±0.19)	2.05 (±0.29)	1.06 (±0.18)	1.37 (±0.33)
*Cp*_R-I1043M	150	1.22 (±0.21) *	2.62(±0.19) *	1.21 (±0.18) *	1.78 (±0.41) *

## Data Availability

Data are contained within the article or Supplementary Materials.

## Supplementary Materials

The following supporting information can be downloaded at: https://www.mdpi.com/article/10.3390/insects13121109/s1, Figure S1: Scanning electron micrographs showing a portion of two cuticle sections of susceptible (A) and resistant (B) pupae; Figure S2: Standard curve for chitin content quantification; Figure S3: Response of *Cx. pipiens* resistant and susceptible strains to DFB bioassay.
