# Supplementary material for: Cuticle Modifications and Over-Expression of the Chitin-Synthase Gene in Diflubenzuron-Resistant Phenotype
Source: Insects. 2022 Nov 30;13(12):1109. doi: 10.3390/insects13121109 (PMC9782986; doi:10.3390/insects13121109)
Supplement: Supplementary file 1 [file insects-13-01109-s001.zip › insects-2019836-supplementary.pdf]

Supplementary Materials

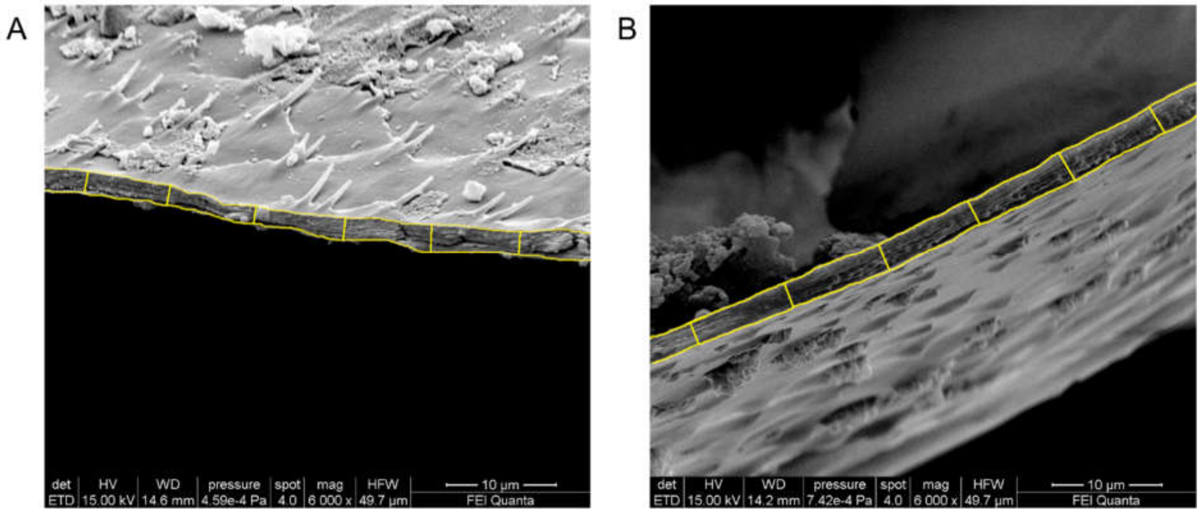

Figure S1. Scanning electron micrographs showing a portion of two cuticle sections of susceptible (A) and resistant (B) pupae. Yellow lines indicate points of cuticle thickness measurement used for calculation of mean cuticle thickness. Thirty points were measured per individual. The image resolution was 10 µm.

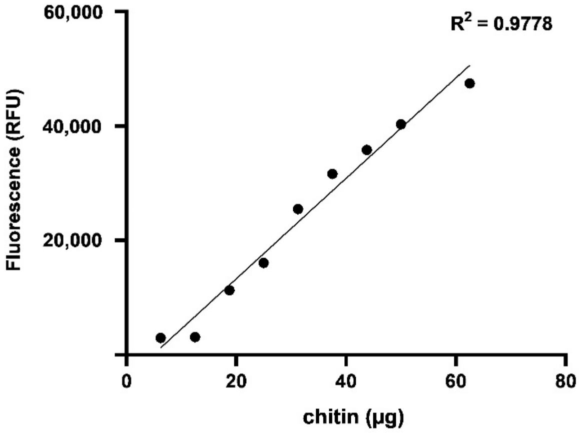

Figure S2. Standard curve for chitin content quantification. The curve was built using increasing volumes of colloidal chitin suspension at 12.5 µg/µl and relating the amount of chitin to the fluorescence intensity of the Calcofluor white fluorescent brightener.

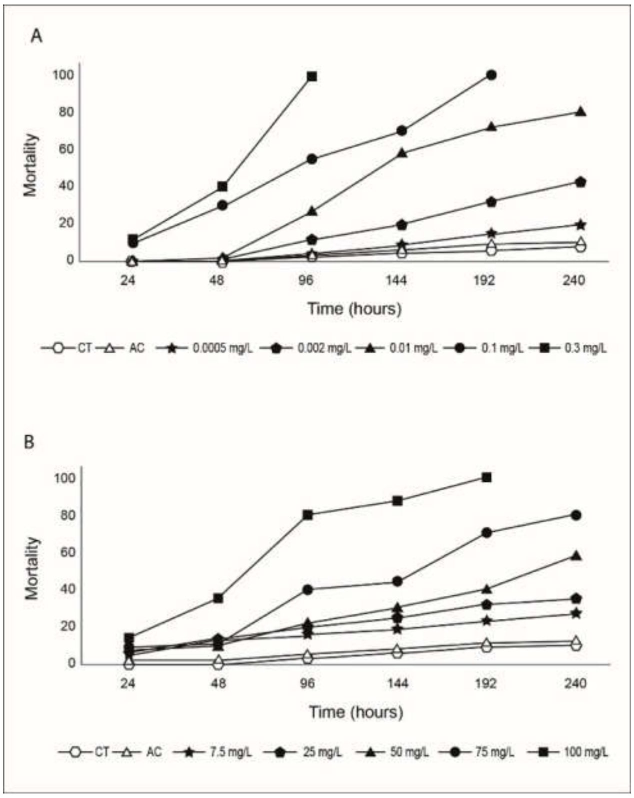

Figure S3. Response of *Cx. pipiens* resistant and susceptible strains to DFB bioassay. Mortality registered in Parma strain (susceptible) (A) and Forlì strain (B) during insecticide exposure. The DFB concentrations used in each strain are also shown. CT = control test with water; AC = control test with acetone. The values represent the mean ± standard error of four replicates.
